# Effect on the Metabolic Biomarkers in Schoolchildren After a Comprehensive Intervention Using Electronic Media and In-Person Sessions to Change Lifestyles: Community Trial

**DOI:** 10.2196/jmir.9052

**Published:** 2018-02-05

**Authors:** Jenny Vilchis-Gil, Miguel Klünder-Klünder, Samuel Flores-Huerta

**Affiliations:** ^1^ Community Health Research Department Hospital Infantil de México Federico Gómez Ciudad de México Mexico; ^2^ Research Committee, Latin American Society for Pediatric Gastroenterology, Hepatology and Nutrition Ciudad de México Mexico

**Keywords:** obesity, child, early intervention (education), insulin resistance, biomarkers

## Abstract

**Background:**

Obesity is a chronic low-intensity state of inflammation with metabolic alterations that, when acquired during childhood, lead to severe illness in adults. Encouraging healthy eating habits and physical activity is the basis for preventing and treating obesity and its complications.

**Objective:**

To evaluate how a comprehensive intervention promoting healthy eating habits and physical activities in schools affects children’s metabolic biomarkers.

**Methods:**

Of four Mexico City primary schools in this study, two groups of children that were recruited at their schools were assigned to a 12-month intervention group (IG) and the other two were assigned to control groups (CGs). The intervention had two components: (1) parents/schoolchildren attended in-person educational sessions promoting healthy eating and physical activity habits, and were provided printed information; and (2) parents were able to seek information through a website, and also received brief weekly mobile phone text messages. Anthropometric measurements and fasting blood samples were taken from both groups of children at baseline and again after 12 months.

**Results:**

The study involved 187 children in the IG and 128 in the CG. Regardless of each child's nutritional status at the beginning of the study, the intervention improved metabolic parameters; the IG showed a negative effect on glucose concentrations (–1.83; CI 95% –3.06 to -0.60), low-density lipoprotein-cholesterol (–2.59; CI 95% –5.12 to –0.06), insulin (–0.84; CI 95% –1.31 to –0.37), and homeostasis model to assess the insulin resistance index (HOMA-IR; –0.21; CI 95% –0.32 to –0.09) in comparison to the CG. HOMA-IR improved in children who had higher than baseline body mass index *z-scores*.

**Conclusions:**

Intervention through multiple components that promoted healthier eating and physical activity habits improved the metabolic parameters of the children in the study after one year, regardless of their nutritional status.

## Introduction

Over the last 25 years, the prevalence of overweight and obese persons has become a worldwide public health issue [1,2]. According to national surveys, the combined incidence of overweight and obese children in Mexico rose from 26.9% in 1999 to 34.8% in 2006, which is one of the highest increments in the world [3]. While surveys from 2012 and 2016 [3] revealed that the problem has not grown in the last ten years (34.4% and 33.2%, respectively), this percentage remains unacceptably high [4].

In recent decades, adults and children have changed their eating and physical activity habits. Foods and beverages have become energy dense and high in saturated fats and refined carbohydrates, and these nutrients present cardiovascular risk factors [5-7]; meanwhile, consumption of fresh fruits, vegetables, and water has diminished [8]. Conversely, both children and adults have become less physically active and spend more time on sedentary activities [9,10].

Childhood obesity is associated with adverse health effects. Obesity is a state of low-intensity chronic inflammation, because adipose tissue produces proinflammatory cytokines that promote insulin resistance and metabolic syndrome [11-13]. These alterations precede the emergence of type 2 diabetes and other cardiovascular diseases [14], which are the most common causes of death. People suffering from obesity who have reduced their body weight have improved their metabolic and inflammatory biomarkers and increased their sensitivity to insulin [15]; however, very few studies have researched how community intervention on nutritional status can affect metabolic parameters.

Blood lipid concentrations in obese children have been positively correlated with blood lipid concentrations in adulthood [16], which is an indication of the long-term risk of these alterations for obese children. When comparing to children who are in a normal weight range, obesity affects blood pressure and blood lipid concentrations and increases insulin resistance [12,17-19]; however, children in a normal weight range can also present metabolic alterations. In a population similar to the one in this study, our research group found that 15% of children with normal weights exhibited low high-density lipoprotein cholesterol (HDL-C) concentrations and 6% had hypertriglyceridemia [11].

Diastolic blood pressure has been positively associated with the consumption of sugary drinks, insulin concentrations have been associated with foods containing refined flour, and triglyceride concentrations have been associated with foods with added fats [8]. Childhood obesity prevention programs promoting healthy eating and physical activity habits have achieved considerable improvements in lipid profiles (low-density lipoprotein cholesterol [LDL-C], HDL-C, and total cholesterol), even in children already in a healthy weight range [20]. These results are likely attributable to better eating habits and increasing physical activity, as opposed to a reduction in adipose tissue. This evidence highlights the importance of introducing prevention programs and healthy lifestyle interventions from an early age.

There are numerous studies in which interventions have aimed to improve metabolic parameters in obese children, mostly through calorie-restriction strategies as opposed to encouraging a change of habits. Conversely, few studies have focused on preventing obesity or target children who (despite being in a healthy weight range) are still at risk [21]. Preventing obesity and its severe health implications requires sustainable strategies. Therefore, elementary schools are an ideal context for providing children and households with trustworthy information regarding the importance of leading a healthy lifestyle [22]. We previously reported that handing parents information on healthy eating and physical activity in person, or sending it through electronic media, can improve their children’s nutritional status [23]. Thus, the objective of this study is to evaluate how an educational intervention aimed at parents can encourage a change of lifestyle, and also modify metabolic biomarkers in schoolchildren.

## Methods

### Design and Study Population

As described by Vilchis-Gil et al [23], this study took place in two public and two private Mexico City elementary schools located in the same geographic area, which were selected for convenience and for their approximately similar number of students. Two of these schools were assigned to intervention activities (intervention group; IG) and two of these schools were used as control groups (CGs). Children of both sexes from grades 1-4 were included in the study. The nutritional status of the children could be eutrophic, overweight, or obese; however, children participating in weight loss programs, suffering from chronic illness, or taking prescription medicine that could affect their metabolic profile were not included. The research protocol was approved by the Hospital Infantil de México Federico Gómez (HIMFG) Research, Ethics, and Biosecurity Committee, and by school authorities. Before initiating the study, students and parents were asked to give written consent and approval. The study was not registered at ClinicalTrials.gov.

### Intervention Implementation

The intervention took place between October 2013 and July 2014; parents were informed of health risks associated with obesity and the benefits of developing healthy eating and physical activity habits. Nutrition and health topics, mobile phone text messages, posters, and other learning materials were prepared in advance.

Long distance activities for parents took place on the website and through parents’ mobile phones. The HIMFG website featured a window where parents could access all project information. The portal, which had several sections, was updated with new topics every 15 days for a total of 20 topics during the entire intervention. Topics included information on how to improve eating and physical activity habits, as well as links to electronic resources providing information on that same topic. Parents were sent weekly text messages of up to 25 words on their mobile phones. Each message encouraged and reinforced behavioral changes and was related to the latest topic on the website. Forty messages were sent in total.

Regarding in-person activities, parents of schoolchildren participating in the intervention attended three hour-long sessions, which were held every two months. Session contents were designed to strengthen their participation in the project and offer opportunities for parents to share their experiences, doubts, and opinions, and offer feedback for the project. Parents were also given two brochures offering information on healthy eating and physical activity.

The children participated in several activities. A team of two nutritionists and a physical education teacher held four bimonthly workshops with a 1.5-hour duration. Workshops incorporated board games, physical games, and learning materials designed to encourage and reinforce healthy eating habits and physical activity. Children were given laminated placemats with images such as the “Healthy Eating Dish” (Plato del Bien Comer) [24] and the physical activity pyramid. The children and their parents visited the Life and Health Balance Hall (Sala Salud Vida en Equilibrio) at Universum Science Museum located at the National Autonomous University of Mexico (UNAM; Universum, Museo de las Ciencias de la UNAM). Every month, posters promoting healthy habits related to the latest topic on the website were placed in visible places around the school, and proved to be very popular among students.

### Sociodemographic Information

Mothers of participating students filled in forms that provided information on their education level and on their child’s sex and age.

### Measurement Anthropometrics

Two nutritionists who were familiar with standardized in international anthropometric procedures [25] measured and weighed the children at baseline and at the end of the study (12 months). Weight was measured on a digital scale (Seca model-882, SECA Corp., Hamburg, Germany) with 0.1 kg precision. Height was measured on a stadiometer (SECA model-225, SECA Corp., Hamburg, Germany) with 0.1 cm precision. Children were measured without shoes and wearing light clothing, standing in the middle of the scale platform or stadiometer, arms resting freely by their sides, with their heads in the Frankfurt horizontal plane.

Body mass index (BMI) *z-scores* were obtained using the children’s age, height, and sex. Children were then classified as underweight (*z-score* < -2), normal weight (*z-score* > -2 to <1), overweight (*z-score* >1 to <2) and obese (*z-score* >2), according to standards provided by the World Health Organization in 2007 [26].

### Biochemical Determinations

Children from both groups gave venous blood samples at baseline and after 12 months, in both cases having fasted for 12 hours. These samples were used to determine glucose, triglycerides, total cholesterol, and HDL-C (ILAB 300, Instrumentation Laboratory, Barcelona, Spain). For LDL-C, we utilized DeLong’s modified Friedwald formula [27]. Insulin was determined by chemiluminescence immunoassay (IMMULITE 2000, Euro, DPC, Llanberis, UK). The following equation was used to obtain homeostasis model to assess the insulin resistance index (HOMA-IR): fasting glucose (mg/dL) x fasting insulin (μU/mL)/405 [28].

### Data Analysis

The study population’s baseline characteristics were described using descriptive statistics. Weight and height measurements were adjusted by age and sex using multiple linear regression. A student’s t-test was used to compare groups’ continuous variables at baseline and the Chi-square test was used for categorical data. The Mann-Whitney U test was used to compare metabolic parameters between groups at baseline.

Subsequently, the analysis was limited to participants whose data was complete at baseline and after 12 months. To evaluate changes in metabolic parameters at baseline and after 12 months, quotients were estimated using quantile regression models, adjusted for dependent variable baseline concentrations, baseline age, sex, and school. After these estimations were determined, the effect of the intervention on the change in metabolic parameters at the end of the study was evaluated. Quantile regression models were used to build two models, the first of which was adjusted for dependent variable baseline concentrations, baseline age, sex, and school; model 2 was adjusted for dependent variable baseline concentrations, baseline age, sex, school, and baseline BMI *z-score*. Finally, we studied the relationship between baseline BMI *z-scores* and changes in HOMA-IR at baseline and after 12 months for both the CG and the IG. *P*-values <.05 were considered significant. Data analysis was completed using STATA SE v.12.0 (Stata Corp, College Station, TX, USA).

## Results

**Figure 1:**
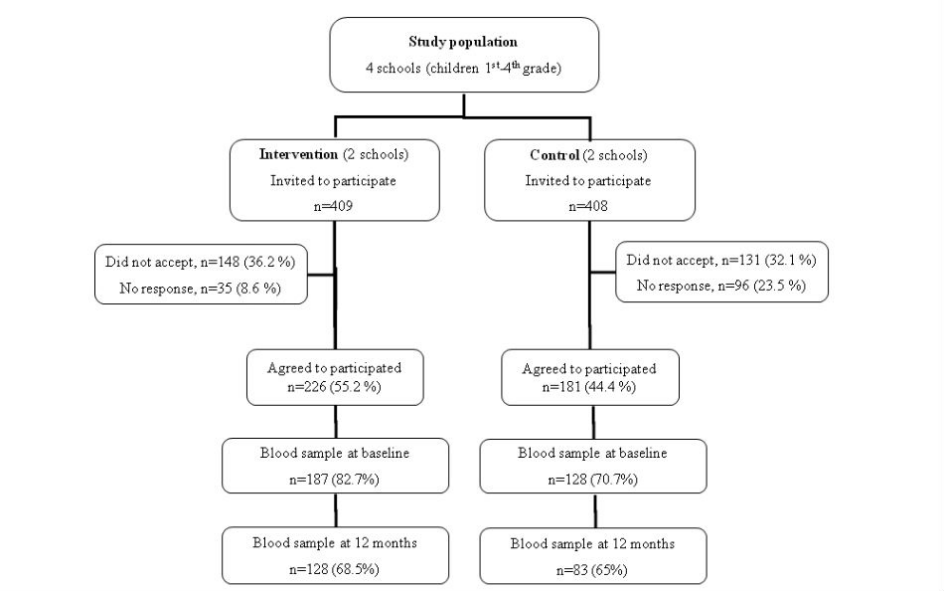
Study population.

Of all the children participating in the study, 82.7% (187/226) of IG children and 70.7% (128/181) of CG children gave baseline blood samples (Figure 1); 68.5% (128/187) of IG children and 64.8% (83/128) of CG children gave blood samples at the end of the 12-month study. Table 1 shows baseline characteristics of the population. Both groups had similar anthropometric and socioeconomic characteristics and the median age was approximately 8 years (standard deviation [SD] 1.2). Data showed that 24.2% (31/128) of CG children and 25.1% (47/187) and IG children were overweight; 28.1% (36/128) of CG children and 21.9% (41/187) of IG children were classified as obese.

Some differences in baseline metabolic parameters were observed between each group (Table 2): the CG presented higher glucose concentrations in comparison to the IG (*P*=.03); while the IG, when compared to the CG, had higher concentrations of total cholesterol (*P*=.001), LDL-C (*P*=.03), insulin (*P*=.001) and HOMA-IR (*P*=.005).

Table 3 shows changes in metabolic parameters between baseline and 12 months for both the CG and IG children. Adjusting for baseline concentrations of each dependent variable, age, sex, and school (model 1), at the end of the intervention, IG children showed reduced glucose concentrations (-1.53; CI 95% -2.66 to -0.40), triglycerides (-5.76; CI 95% -9.90 to -1.62), total cholesterol (-3.36; CI 95% -6.66 to -0.05), LDL-C (-2.56; CI 95% -4.89 to -0.22), insulin (-0.97; CI 95% -1.54 to -0.40), and HOMA-IR (-0.22; CI 95% -0.34 to -0.11). Model 2 shows that regardless of children’s BMI *z-score* at the start of the study, the intervention had beneficial effects on participants’ metabolic parameters.

**Figure 2:**
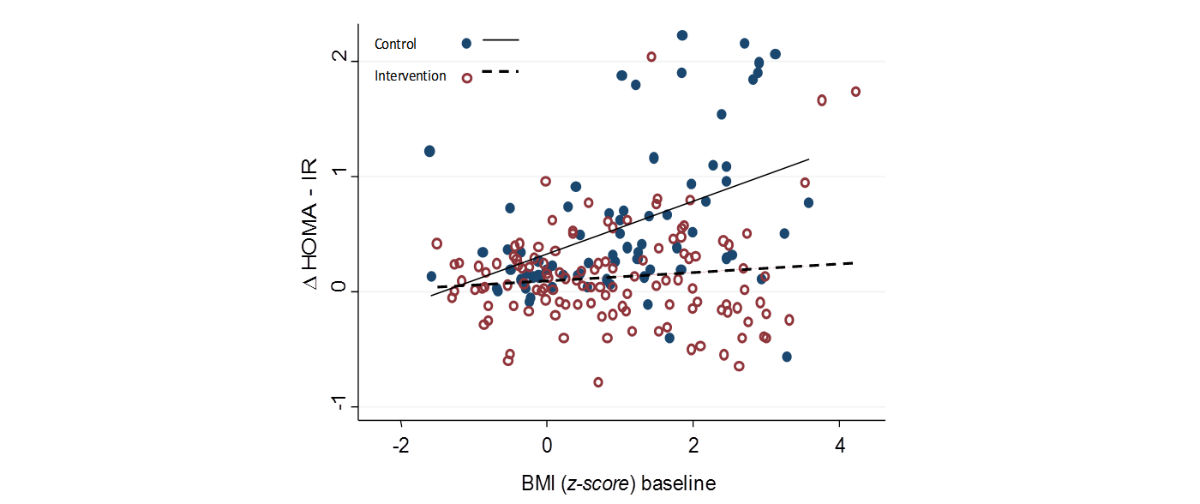
Change of baseline at 12 months in the homeostasis model assessment-estimated insulin resistance (HOMA-IR) and the relationship with body mass index (BMI) z-score baseline in the groups.

Figure 2 shows the relationship between changes in HOMA-IR at baseline and after 12 months, and children’s BMI *z-scores* at baseline. HOMA-IR changes at the end of the study showed differences between study groups. Children in the CG showed an increase, which was higher when baseline BMI *z-scores* were higher, while children in the IG maintained stable HOMA-IR regardless of their BMI *z-score*.

**Table 1:** Baseline characteristics of the study population (n=315).

Characteristic		Control (n=128)	Intervention (n=187)	*P* value^a^
Age in years, mean (SD)		8.1 (1.2)	7.9 (1.2)	.26
Sex (female), n (%)		70 (54.7)	85 (45.5)	.11
**Anthropometric, mean (SD)**				
	Weight (kg)^b^	30.4 (5.0)	29.7 (5.0)	.26
	Height (cm)^b^	127.5 (7.1)	126.6 (7.1)	.30
	Body mass index (BMI) *z-score*^c^	1.07 (1.3)	0.94 (1.4)	.41
**Classification of BMI *z-score***^a^**, n (%)**			
	Normal weight (*z-score* > –2 to <1)	61 (47.7)	99 (52.9)	
	Overweight (*z-score* ≥1 to <2)	31 (24.2)	47 (25.1)		
	Obesity (*z-score* ≥2)	36 (28.1)	41 (21.9)	.44
**Maternal schooling, n (%)**				
	Middle school or less	15 (12.1)	34 (19.4)	
	High school or technical school	55 (44.4)	67 (38.3)	
	College career or postgraduate	54 (43.5)	74 (42.3)	.22
**School, n (%)**				
	Public	63 (49.2)	112 (59.9)	
	Private	65 (50.8)	75 (40.1)	.06

^a^*P* value according to Student´s *t* test and χ2 test.

^b^Means adjusted by age and sex.

^c^World Health Organization standard, 2007.

**Table 2:** Metabolic parameters by study group at baseline.

Parameter	Control (n=128) Median (Q1-Q3)	Intervention (n=187) Median (Q1-Q3)	*P* value^a^
Glucose (mg/dL)	87 (82-93)	85 (81-90)	.03
Triglycerides (mg/dL)	67 (54-90)	73 (49-102)	.32
Total cholesterol (mg/dL)	155 (143-175)	170 (150-185)	.001
LDL-C^b^ (mg/dL)	92.9 (81.7-110.3)	103.0 (85.4-117.4)	.03
HDL-C^c^ (mg/dL)	47 (39-55)	50 (41-57)	.15
Insulin (μU/mL)	2.6 (1.9-5.1)	3.8 (2.2-6.6)	.001
HOMA-IR^d^	0.53 (0.43-1.21)	0.79 (0.46-1.39)	.005

^a^*P*-value according Mann-Whitney *U* test.

^b^LDL-C: low-density lipoprotein cholesterol.

^c^HDL-C: high-density lipoprotein cholesterol.

^d^HOMA-IR: homeostasis model to assess the insulin resistance index.

**Table 3:** Change in metabolic parameters from baseline to 12 months.

Parameter^a^	Control (n=74) change 0-12 months (95% CI)^b^	Intervention (n=119) change 0-12 months (95% CI)^b^	Intervention effect, model 1, β (95% CI)^c^	*P* value	Intervention effect, model 2, β (95% CI)^d^	*P* value	
Glucose (mg/dL)	4.86 (3.84 to 5.88)	3.38 (2.79 to 3.98)	-1.53 (–2.66 to –0.40)	.008	–1.83 (–3.06 to –0.60)	.004
Triglycerides (mg/dL)	2.25 (–1.16 to 5.67)	–6.74 (–9.20 to –4.29)	-5.76 (-9.90 to -1.62)	.007	-5.25 (–1.46 to 0.97)	.01
Total cholesterol (mg/dL)	1.76 (–1.10 to 4.62)	-2.54 (–6.38 to 1.31)	-3.36 (–6.66 to –0.05)	.046	-3.22 (–7.11 to 0.67)	.10
LDL-C (mg/dL)	1.10 (–1.74 to 3.93)	-1.62 (–3.96 to 0.72)	-2.56 (–4.89 to –0.22)	.03	-2.59 (–5.12 to –0.06)	.045
HDL-C (mg/dL)	-0.47 (–1.31 to 0.37)	-0.14 (–0.74 to 0.49)	0.55 (–0.54 to 1.63)	.32	0.36 (–0.73 to 1.45)	.52
Insulin (μU/mL)	1.30 (0.60 to 1.99)	–0.09 (–0.29 to 0.11)	–0.97 (–1.54 to –0.40)	.001	-0.84 (–1.31 to –0.37)	.001
HOMA-IR	0.37 (0.16 to 0.58)	0.03 (–0.01 to 0.06)	-0.22 (–0.34 to –0.11)	<.001	–0.21 (–0.32 to –0.09)	.001

^a^LDL-C: low-density lipoprotein cholesterol; HDL-C: high-density lipoprotein cholesterol; HOMA-IR: homeostasis model to assess the insulin resistance index.

^b^Change from baseline at 12 months, adjusted for baseline data of the dependent variable, baseline age, and sex.

^c^Quantile regression models, adjusted for baseline data of the dependent variable, baseline age, sex, and public and private school.

^d^Quantile regression models, adjusted for baseline data of the dependent variable, baseline age, sex, public and private school, and baseline BMI *z-score.*

## Discussion

This study shows that an educational intervention focused on promoting healthy eating and physical activity habits aimed at parents and their children, through in-person and long-distance activities, can improve children’s metabolic parameters regardless of their baseline nutritional status. Some studies have shown that diets low in saturated fats and rich in fruits and vegetables reduce the risk of cardiovascular disease [29], while an increase in physical aerobic activity reduces insulin levels regardless of weight loss [30]. Childhood overweight and obesity prevention programs promoting healthy eating and physical activity habits, in children of any nutritional status, have proven to significantly improve lipid profiles in children (LDL-C, HDL-C, and total cholesterol), including children who are within a healthy weight range [20,31,32]. Conversely, a meta-analysis has shown that interventions targeting multiple components (eg, diet and/or physical activity) in family or school contexts that improve body adiposity measurements (BMI, BMI *z-score*) also improve LDL-C, HDL-C, and triglycerides, as well as insulin sensitivity, while studies not targeting body adiposity measurements did not improve these factors [20]. Previously published anthropometric data from this study [23] showed that children in the IG, especially those suffering from obesity, reduced their BMI *z-scores*. In this study, greater HOMA-IR changes were observed in children presenting obesity; for this reason, changes in metabolic parameters reported here may be associated with a change in children’s nutritional status and/or changes in eating and physical activity habits. It remains to be determined how long the effects of an educational intervention can last on metabolic parameters.

It is known that alterations in lipid profiles, insulin, and HOMA-IR during childhood are risk factors for cardiovascular diseases and generally carry over into adulthood [33,34]. Studies have shown that insulin and HOMA-IR levels increase during puberty [33]. In a study of healthy children by prospective cohort (EarlyBird) insulin resistance and HOMA-IR increased in a linear fashion as of age 7, and even before puberty [33]. This increase could be due to an increase in adiposity as of this age (and fat inhibiting the effect of insulin), or a progressive increase in insulin-like growth factor-1 that takes place as puberty approaches and has effects associated with insulin resistance [33,34]. In this study, in-person and distance learning programs focused on improving eating habits and physical activity proved to halt this possible age-related increase of insulin concentrations and HOMA-IR in the IG. Insulin resistance is considered an important physiopathological factor that underlies many complications that result from childhood obesity [16].

The change in the HOMA-IR biomarker found in this study is less significant than the findings of other studies [35]; this may be due to the changes achieved in the present study within a year of intervention, which included children who were of healthy weight, who were overweight, and who were obese, while studies with higher changes in HOMA-IR were focused on calorie restriction [36] and were directed specifically at children suffering from obesity [21,36,37]. However, it should be highlighted that in the present study, the effects on metabolic parameters were greater in obese children. Although small changes achieved in one year are important when improving lifestyles, a steady increase in unhealthy habits over time can contribute to the development of cardiovascular disease. Given the magnitude of the problem, individual treatment is unsustainable and low-cost community-wide measures that reach a greater number of people and promote better eating habits and physical activity in families become necessary [38]. This intervention sought the participation of parents, which is an important factor to improve adherence to changes in eating and physical activity habits in children.

Excess adipose tissue contributes to a chronic state of inflammation and increases insulin resistance, along with other metabolic complications [39]. Programs encouraging healthy eating and physical activity should be promoted among the population as a way of improving lipid profiles and preventing or reverting insulin resistance, as well as preventing chronic diseases such as metabolic syndrome, diabetes, and other comorbidities.

It is important to highlight that this study had certain limitations. Schools were not randomly assigned to study groups, and data on changes in eating habits and physical activity, which would explain changes in metabolic parameters, has not been presented. However, the intervention on multiple components also had some strengths, such as: (1) parents were treated as a household proxy, which is where children obtain their habits; (2) the study enabled parents to use their time more efficiently in this very complex city; (3) the study followed participants for 12 months, which is more time than other studies [21]; and (4) the use of technological resources was an innovative way to answer the problem of overweight and obese children.

Finally, our data indicates that strategies that imply adiposity reduction are likely to succeed in modifying lipid profiles and insulin resistance, which are sensitive to changes in body composition. However, these parameters are also independently sensitive to changes in diet and physical activity in children. This evidence highlights the importance of introducing early prevention programs and interventions aimed at promoting healthy lifestyles with the purpose of preventing adverse health effects in adult age. In conclusion, results found in this study suggest that educational interventions using electronic media and in-person sessions to promote healthy eating and physical activity habits improved children’s metabolic parameters, and especially benefitted glucose metabolic parameters, independently of children’s nutritional status at the start of the study.

## Appendix

Multimedia Appendix 1 CONSORT‐EHEALTH checklist (V 1.6.1).

## Abbreviations

BMI: body mass index
CG: control group
HDL-C: high-density lipoprotein cholesterol
HIMFG: Hospital Infantil de México Federico Gómez
HOMA-IR: homeostasis model to assess the insulin resistance index
IG: intervention group
LDL-C: low-density lipoprotein cholesterol
UNAM: National Autonomous University of Mexico
